# Exploring the spatio-temporal clusters of closed restaurants after the COVID-19 outbreak in Seoul using relative risk surfaces

**DOI:** 10.1038/s41598-023-40937-5

**Published:** 2023-08-24

**Authors:** Sungjae Park, Hyunil Seo, Hyeongmo Koo

**Affiliations:** The Department of Geoinformatics, University of Seoul, Seoul, 02504 Republic of Korea

**Keywords:** Environmental social sciences, Environmental economics, Environmental impact

## Abstract

This study explores the clusters of closed restaurants in Seoul in response to the COVID-19 pandemic using the relative risk surface (RRS). The RRS developed based on kernel density estimation provides alternative perspectives for finding the cluster by combining different control and case events. Specifically, the varying impacts on diverse types of restaurants are examined by comparing the densities of closed casual restaurants and cafes. The clusters of closed businesses following the COVID-19 outbreak are subsequently explored through a comparison of the densities of the closed businesses preceding the outbreak. Furthermore, this analysis estimates the clusters of declined commercial areas after the pandemic outbreak based on the comparison between the densities of opened and closed restaurants. Finally, the specific time and region of the clusters are explored using space–time RRS. The analysis results effectively demonstrate various aspects of the closed restaurant clusters. For example, in the central business areas, the densities of closed cafes have decreased after the pandemic outbreak, and the density of closed cafes is significantly higher than that of opened cafes. This study would contribute to the literature on spatial data analysis and urban policy support in response to future epidemics.

## Introduction

The Coronavirus disease (COVID-19) has spread to an unprecedented pandemic worldwide. As of 2022, the COVID-19 pandemic has globally surpassed five hundred million of confirmed incidences. Korea has been also suffered from the pandemic with the first case reported in Daegu in February 2020. From the second half of 2020, Seoul became the epicenter of the pandemic in Korea with the highest number of confirmed.

The Korean government has implemented social distancing measures that is known to as the effective COVID-19 countermeasure to mitigate the spread of the pandemic and reduce the number of confirmed cases^1^. From February 29, 2020, to May 16, 2022, the measure included restrictions on business hours and private gatherings, and recommendations for telecommuting and non-face-to-face classes in schools. However, the measure has also resulted in a significant reduction in population movement, which could have negative impacts on the sales of local businesses^2–4^.

Exploring clusters of business closings in difference perspectives (e.g., in response to COVID-19, types of business, and declines in commercial districts) can facilitate the development of locally specific policy supports to assist the regions that are suffered from the social distance measures. In Seoul, small business owners that could not overcome decreases in sales due to prolong the social distancing policy comprise a significant proportion of the industries^5^. Continued decreases in sales could result in the closure of these small businesses, ultimately contributing to a decline in the region^6^ and reduced tax revenue. To prevent business closures, local governments may provide subsidies to affected business owners. Therefore, identifying specific clusters of business closures can provide base research for policymakers to develop effective policy interventions.

Kernel density estimation (KDE) has widely utilized in various fields for exploring spatial clusters of point events^7,8^. Previous studies have also applied KDE to explore clusters of COVID-19 incidences^9,10^, and moreover, space–time kernel density estimation (STKDE) has been utilized to identify clusters in both spatial and temporal dimension of events^11^. While both KDE and STKDE can effectively identify clusters, these clusters may reflect only the population at risk of events when the events are closely related to their population at risk^7,12^. For example, the incidence of infectious diseases in a region is generally related to the population size of that region.

Relative risk surface (RRS) and its extensions provide an alternative method for discovering different types of clusters, which could be useful for exploring clusters of closed businesses after the COVID-19 pandemic. RRS was initially developed to find statistically significant spatial clusters by comparing the densities of point events to that of their population at risk^12,13^. RRS can be extended to various pairs of case and control densities, providing additional insight into cluster exploration. For example, it can explore temporal increases and decreases in case densities compared to corresponding control densities^7,14^. RRS identifies clusters of closed businesses resulting from the COVID-19 pandemic by comparing the densities of closed businesses before and after the pandemic outbreak. Moreover, space–time RRS (STRRS) emphasizes the region and specific time of spatio-temporal clusters^7,15^. Although the clusters RSS finds may not directly result from the pandemic, they can provide valuable exploratory data analysis results facilitating further investigations.

The purpose of this study is to explore clusters of closed businesses, with a focus on restaurants, in Seoul, Korea, following the COVID-19 pandemic. Given that the restaurant industry is a major sector in Seoul, this study extends RRS to explore various clusters of closed restaurant businesses. Specifically, this analysis compares the impacts on different types of restaurants and identify clusters of closed businesses following the COVID-19 outbreak by comparing the densities of closed businesses before and after the outbreak. Additionally, RRS estimates clusters of declined commercial areas after the outbreak by comparing the densities of opened and closed restaurants. Finally, this study uses STRRS to explore the specific time of the clusters.

## Method

### (1) Data and study area

This study identifies clusters of restaurant closures in Seoul following the COVID-19 pandemic outbreak using RRS and its extensions. According to the Korean 2020 census, Seoul has a population of approximately 9.6 million residents and is considered the most densely populated city in Korea. In addition, Seoul had the highest number of confirmed COVID-19 cases in Korea following the outbreak. Given the high population density and incidence rates in Seoul, the COVID-19 pandemic and subsequent social distancing policies have had a significant impact on local businesses.

This study examines the impact of the COVID-19 pandemic on local businesses with a focus on the restaurant industry. This industry was selected for the following analysis due to its significant contribution to the overall economy in Seoul and the negative impacts that it has experienced from the pandemic^3^. Specifically, according to the 2020 business survey conducted by Statistics Korea, the restaurant and lodging sector comprise the second highest proportion of the industries, accounting for about 16% of the total number of businesses. Additionally, the Seoul Institute reports a substantial decrease of 11% in restaurant industry sales in Seoul following the outbreak.

The impact of the pandemic on restaurant businesses may vary based on the type of restaurant. This study categorizes restaurant businesses into two major types, casual restaurants (e.g., Korean food restaurants, family restaurants, and foreign food restaurants) and cafes (e.g., coffee shops, fast food restaurants, and food trucks), based on the Korean food hygiene law. To create restaurant opening and closure events, data on dates and geographic coordinates are obtained from the registration information provided by local administrative agencies (LOCALDATA, https://www.localdata.go.kr/main.do, accessed on 1 March 2023).

### (2) KDE and STKDE

This study employs RRS and its extensions to identify clusters of closed restaurants. Given that RRS is founded on KDE, this section starts with a brief review of KDE. KDE is an effective method for exploring clusters of point events by visually representing their local density in space^16^. KDE can be expressed as follows:

$$\widehat{f}\left(\mathbf{s}\right)=\frac{1}{n{h}_{s}^{2}}{\sum }_{i=1}^{n}{k}_{s}\left(\frac{\mathbf{s}-{\mathbf{s}}_{i}}{{h}_{s}}\right)$$

where $n$ is the number of events, ${h}_{s}$ is the spatial bandwidth, ${k}_{s}$ is the spatial kernel density function, and $\mathbf{s}-{\mathbf{s}}_{i}$ is the distance between event $i$ and kernel center.

STKDE estimates spatio-temporal densities by incorporating a temporal kernel function to estimate the temporal dimension under the first-order separable assumption between space and time^17,18^:

$$\widehat{f}\left(\mathbf{s},t\right)=\frac{1}{n{h}_{s}^{2}{h}_{t}}{\sum }_{i=1}^{n}{k}_{s}\left(\frac{\mathbf{s}-{\mathbf{s}}_{i}}{{h}_{s}}\right){k}_{t}\left(\frac{t-{t}_{i}}{{h}_{t}}\right)$$

where ${h}_{t}$ is the temporal bandwidth, ${k}_{t}$ is the temporal kernel density function, and $t-{t}_{i}$ is the time difference between event $i$ and kernel center.

KDE cannot be determined whether clusters are deviated from a random chance occurred from their population at risk^19^. Specifically, KDE is effective in identifying clusters when the underlying population risk for events is uniformly distributed. However, in reality, the underlying population risk is often non-uniformly distributed because the underlying spatio-temporal processes of individual events vary across space and time^7^. For example, when comparing the densities of confirmed COVID-19 incidents between urban and rural areas, it is necessary to consider the populations at risk of COVID-19 incidence in each area. Since urban areas typically have larger populations than rural areas, the higher number of confirmed incidents in urban areas is simply a reflection of the higher population size.

### (3) RRS and STRRS

RRS extends KDE to examine the statistical significance of case event densities in relation to their corresponding population at risk (i.e., control event densities)^20^. RRS generates KDEs for both case and control events, facilitating a direct comparison of the two KDEs. A logarithm form is generally recommended to mitigate the impacts of extreme values and to improve the symmetry of its estimated values^21^:

$$\rho \left(\mathbf{s}\right)=\mathrm{ln}\frac{{\widehat{f}}_{case}(\mathbf{s})}{{\widehat{g}}_{control}(\mathbf{s})}$$

where ${\widehat{g}}_{control}(\mathbf{s})$ and ${\widehat{f}}_{case}(\mathbf{s})$ are KDEs of control and case events, respectively.

The selection of proper case and control events is a crucial step in RRS, which has the potential to detect extended clusters using various case and control combinations. RRS initially compares the densities of case events to that of their population at risk^12^, but it can be extended to various pairs of case and control densities^14^. This study employs three different case and control pairs to broaden the understanding of the negative impacts of the COVID-19 pandemic on restaurants businesses. First, this study compares the densities of closed casual restaurants and cafes to estimate the varying impacts on different types of restaurant businesses. Second, the densities of closed businesses before (2018–2019) and after (2020–2021) the pandemic outbreak are compared to explore the changes in restaurant closure densities. Third, the comparison between the densities of open and closed restaurants is conducted to estimate the decline in commercial districts. Although comparing closed and ongoing restaurants that are currently operated may be more appropriate, this study chose to use newly opened restaurants, instead of ongoing restaurants, to emphasize the possibility of rehabilitation of restaurant businesses^22^.

Both RRS and STRRS are utilized to conduct the third comparison, which involves comparing the densities of closed restaurants to those of opened restaurants. Similar to the relationship between RRS and KDE, STRRS is developed based on STKDE. STRRS compares STKDEs of control and case spatio-temporal events as follows^23^:

$$\rho \left(\mathbf{s},t\right)=\mathrm{ln}\frac{{\widehat{f}}_{case}(\mathbf{s}, t)}{{\widehat{g}}_{control}(\mathbf{s}, t)}$$

where ${\widehat{g}}_{control}(\mathbf{s}, t)$ and ${\widehat{f}}_{case}(\mathbf{s}, t)$ are STKDE of control and case events, respectively.

### (4) Parameter setup

RRS and STRRS provide a statistically significant test for identifying spatio-temporal clusters based on the permutation test^7,24^. The permutation test compares the densities of case and control events and assesses the statistical difference between them. Specifically, the test randomly shuffles case and control events, and RRS and STRRS are estimated based on the shuffled events. This procedure is repeated 999 times, and *p*-values are computed based on the estimated reference distribution of RRS and STRRS values. The significance level for each cell should be inflated to avoid the multiple-testing problem^25,26^. However, this requires extensive increases in the number of permutations, and thus, this study could not apply the multiple-testing corrections (e.g., Bonferroni and false discovery rate adjustments). The simulated values can also be utilized for a global clustering test by comparing the sums of squared values^27^, which assesses whether the densities of case events are consistent with those of control events across a study area.

The selection of bandwidth sizes and kernel functions plays a crucial role in both RRS and STRRS, similar in KDE and STKDE. The size of the bandwidth is the most critical component because it determines the degree of smoothness in the estimated density surfaces^8^. Generally, a larger bandwidth size generates a smoother surface, but it may not adequately capture local variations in the estimated density surface. In contrast, smaller bandwidth size produces a spiky surface, which makes it difficult to visualize the global trends of an event distribution. This study estimates the optimal bandwidth size using the plug-in method^28^ based on the pooled standard deviations of control and case events, while also adjusting the estimated size for improved intuitive understanding. For RRS and STRRS, using the same bandwidth size for case and control data is recommended to maintain the same spatial and temporal ranges^29^.

The selection of kernel functions also influences the smoothness of estimated density surfaces, although this choice is less critical than the selection of bandwidth size^13^. This study employs a Gaussian density function to highlight the general trend of closed restaurant businesses because this function generates smoother surfaces than other kernel functions (e.g., quartic and triweight functions)^30^. Additionally, this study addresses underestimations of RRS and STRRS at the spatio-temporal edges of the study areas in the following ways. Spatial edge effects are corrected using Berman & Diggle’s method^31^, and temporal edge effects are adjusted by creating a toroidal wrap^32^.

## Result

This section presents the analysis results of the closed restaurants (i.e., casual restaurants and cafes) clusters in response to the COVID-19 pandemic. First, the study provides a general perspective on the spatio-temporal distribution of the number of closed restaurants using a histogram and KDE. Next, this study examines changes in the densities of closed restaurants after the pandemic outbreak using RRS. Second, this study estimates RRS clusters obtained from various pairs of case and control densities. Specifically, it examines the varying impacts on different types of restaurant businesses by comparing the densities of closed casual restaurants and cafes. Furthermore, changes in the densities of closed businesses before and after the outbreak are explored by comparing the densities of closed businesses before and after the outbreak. Finally, this study estimates declined commercial areas after the pandemic outbreak using RRS and STRRS by comparing the densities of closed restaurants to those opened restaurants.

### (1) The spatio-temporal distributions in closed restaurants

**Figure 1:**
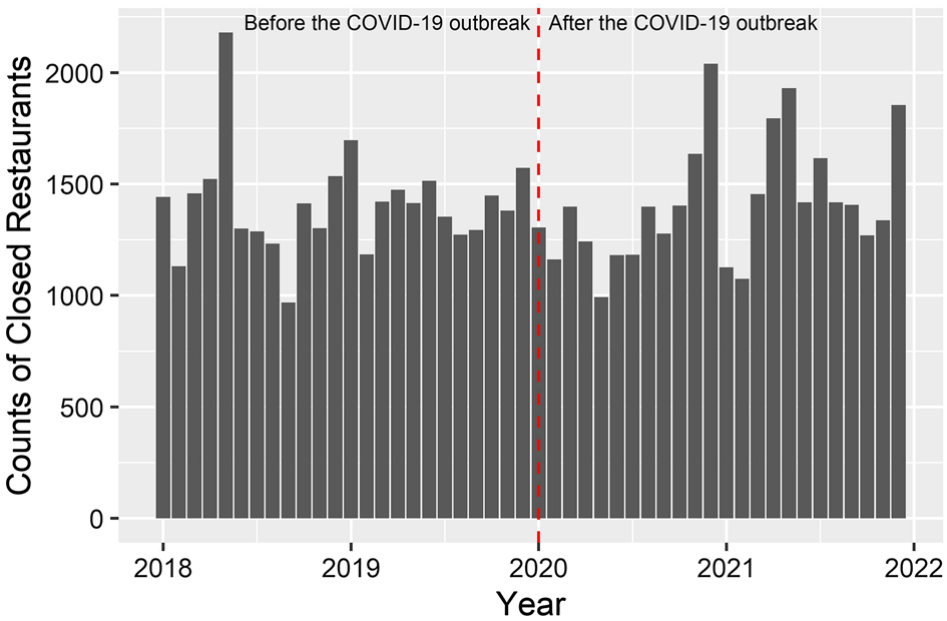
A time series histogram for the number of closed restaurants with 1 month interval.

Figure 1 shows the temporal changes in the number of closed restaurants at monthly intervals. A total of 67,717 restaurants had closed during the 4-year period from 1 January 2018 to 31 December 2022. The numbers of closed restaurants are 33,796 and 33,921 before and after the COVID-19 outbreak, respectively, and they show temporal variations (Fig. 1). Similar to these temporal variations, spatial variations in the distribution of closed restaurants might also exist.

**Figure 2:**
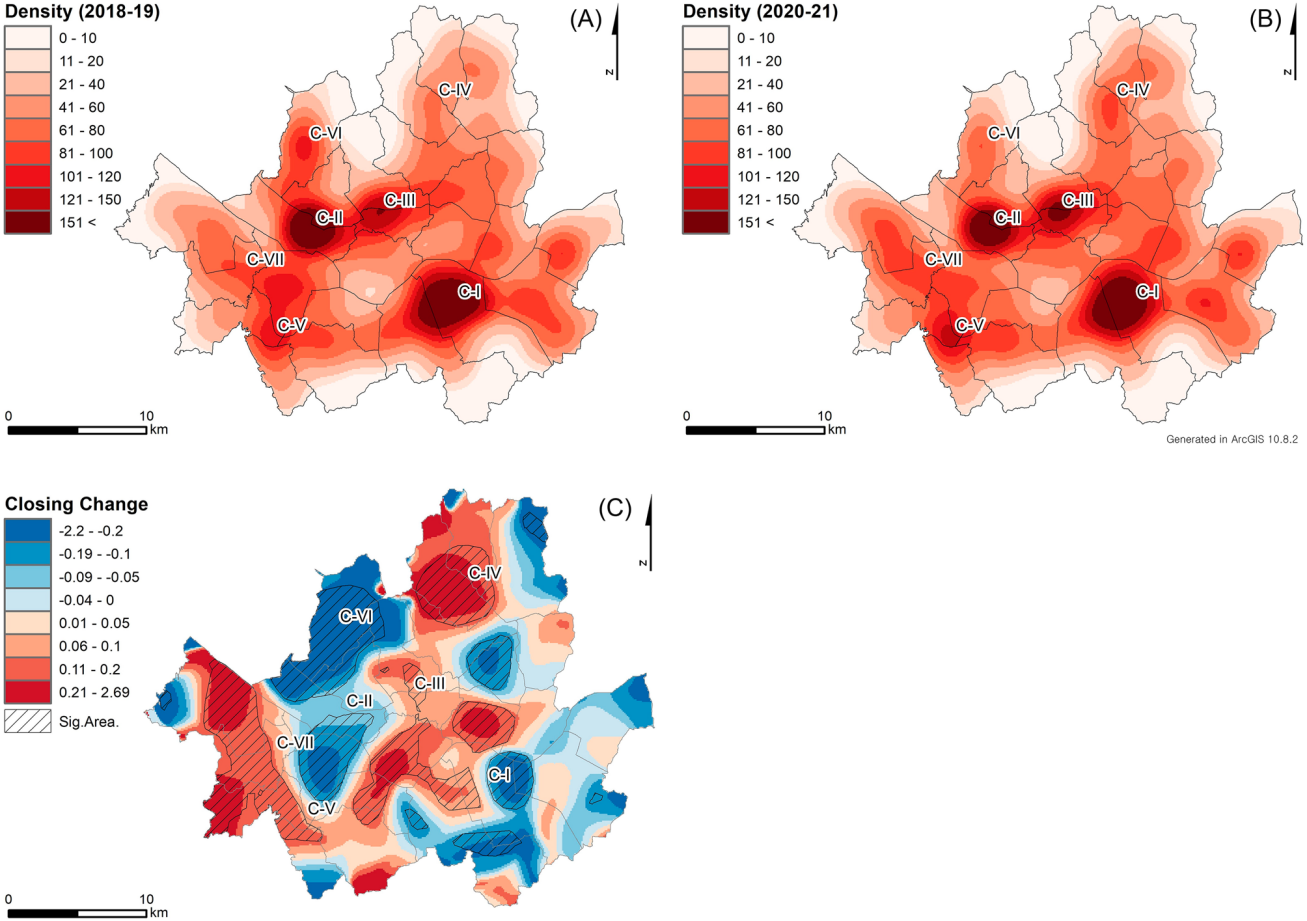
The KDE results of closed restaurants (**A**) before and (**B**) after the pandemic and (**C**) the RRS results by comparing them.

Figure 2 presents the spatial clusters of closed restaurants resulting from the COVID-19 pandemic. The clusters of interest in this study are sequentially numbered from C-I to C-VII for reference purposes. Figure 2A, B display KDE results of closed restaurants during the periods of 2018–2019 and 2020–2021, respectively. The bandwidths for the KDEs are estimated to 1050 m based on the plug-in methods, but they are adjusted to 1000 m to enhance intuitive understanding and ease of analysis. The KDE results reveal three major clusters (i.e., C-I, C-II, and C-III) in central business and major commercial districts in Seoul, as well as four minor clusters (i.e., from C-IV to C-VII) in local commercial districts.

The KDE results (Fig. 2A, B) demonstrate changes in the size and shape of the clusters following the COVID-19 outbreak. Notably, clusters C-III, C-IV, C-V, and C-VII have emerged after the outbreak, whereas the sizes of clusters C-II and C-VI have decreased. The shape of cluster C-I has also changed slightly from an east–west orientation to a north–south orientation. Cluster C-III, located in the central business district (CBD) and containing major tourist attractions such as Myeongdong in Seoul, has experienced a significant increase in size (Refer to the Supplementary Figure). This may be due to the implementation of strict social distancing policies such as telecommuting and allowing restaurants and cafes to provide only delivery and takeout services, as well as travel restrictions for foreigners^33^.

The RRS result (Fig. 2C) highlights changes in local clusters of closed restaurant densities in response to the COVID-19 pandemic, although the global clustering test does not show a statistical difference in closed restaurant densities after the outbreak (*p*-value of 0.307 with 999 permutations). The areas with line fill symbols indicate statistically significant local clusters of changes in closed restaurant densities based on the permutation test. Positive RRS values in C-III, C-IV, C-V, and C-VII with line fill symbols indicate significant increases in closed restaurant densities after the COVID-19 outbreak. This highlights the increase in C-III (i.e., the CBD) due to social distancing policies. Negative RRS values in C-I, C-II, and C-VI indicate that the densities of closed restaurants have decreased, although they still show relatively high densities of closed restaurants after the outbreak. The significant area in C-I successfully captures the changes in its shapes of C-I (Fig. 2A, B).

### (2) Comparing closed restaurant densities before and after the pandemic outbreak

**Figure 3:**
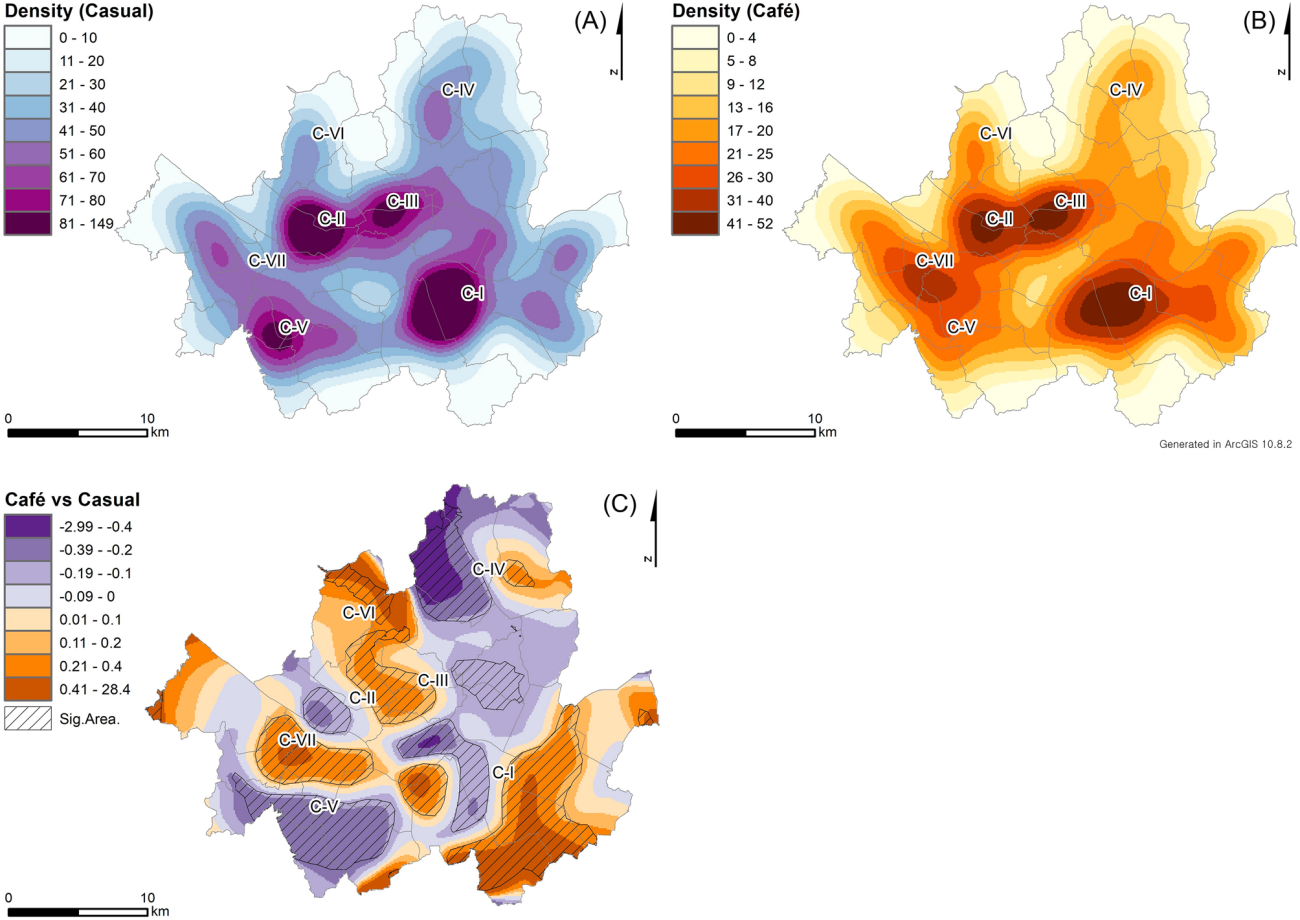
The KDE results of closed (**A**) casual restaurants and (**B**) cafes with (**C**) the RRS results by comparing (**A**) and (**B**) as control and case, respectively.

The COVID-19 pandemic has had varying impacts on different types of restaurants, specifically casual restaurants and cafes, as shown in their KDE results (Fig. 3A, B). The bandwidth size for both KDEs is estimated to 1250 m using the plug-in method based on the pooled standard deviations of all restaurants. These results differ slightly from those of all restaurants (Fig. 2), thus requiring further explanation of the clusters of closed restaurants based on their types. The KDE values for closed casual restaurants are relatively higher in C-I, C-II, C-III, and C-V, whereas those for cafes are higher in C-I, C-II, C-III, and C-VII. The clusters are commonly found in C-I, C-II, and C-III, which are the CBD and major commercial districts. The café KDE result emphasizes C-VII, which consists of a newly developed café area (e.g., Munrae-Dong), while the casual restaurant KDE result highlights C-V, where business offices are concentrated (e.g., Gasan digital complex) (Refer to the Supplementary Figure).

The impacts of the pandemic on restaurant businesses need to be analyzed by considering the different types of restaurants as the RRS result (Fig. 3C) explicitly shows the varying impacts on casual restaurants and cafes. This analysis uses RRS to compare the KDE results of closed casual restaurants and café (Fig. 3A, B). The global clustering of the RRS results supports their statistical difference with the *p*-value of 0.001. The RRS analysis was conducted using the same bandwidth size as its corresponding KDE results. Additionally, the RRS result shows local variations in the clusters of closed restaurants, with significantly different areas marked using line fill symbols. Specifically, positive RRS values indicate that the densities of closed cafes are relatively higher than those of closed casual restaurants, while negative RRS values indicate that closed casual restaurants are dominant in the corresponding areas. The results clearly show that café closings are dominant in areas C-VII, while casual restaurant closings are more prevalent in areas C-V.

**Figure 4:**
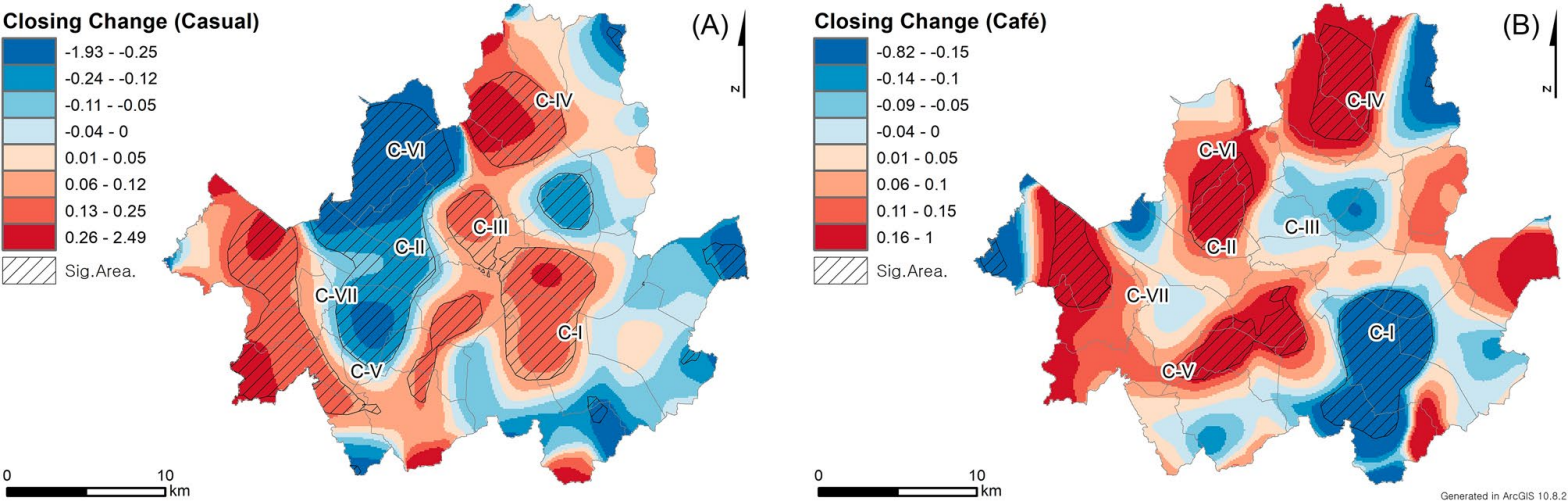
The RRS results by comparing the densities of closed (**A**) casual restaurants and (**B**) cafes before and after the pandemic outbreak.

The spatial distributions of closed casual restaurants and cafes after the COVID-19 pandemic outbreak exhibit distinct differences (Fig. 4), which provides additional insights into the patterns of closed restaurants. Specifically, the RRS analyses are conducted for each restaurant type by comparing the KDE results of closed restaurants after (Fig. 3A, B) and before the pandemic outbreak. Overall, the RRS result of closed casual restaurants (Fig. 4A) is more similar to that of all closed restaurants (Fig. 2C) because the number of closed casual restaurants is higher than that of cafes. The RRS results of casual restaurants and all closed restaurants only differ in C-I and C-III. Specifically, for casual restaurants, C-I exhibits positive RRS values, indicating an increase in the density of closed casual restaurants after the outbreak. In C-III, the cluster sizes of the positive RRS values are larger for casual restaurants than for all closed restaurants. These differences lead to a significant result in the global clustering test, with a *p*-value of 0.018, indicating a statistically significant difference in closed casual restaurants after the outbreak, while no significant difference was observed in the analysis of all closed restaurants.

The RRS results for closed cafes represent distinct spatial patterns compared to those of closed casual restaurants. While the RRS values for closed cafes in C-IV, C-V, and C-VII show relatively similar patterns to those for closed casual restaurants, the RRS values for C-I, C-II, C-III, and C-VI indicate opposite trends between closed cafes and casual restaurants. Specifically, the densities of closed cafes are higher in C-I and C-III (Fig. 3B), but they have decreased after the pandemic outbreak. However, the densities of closed cafes are higher in C-II, where the RRS result shows an increase in densities after the outbreak. Moreover, the RRS result indicates significant changes in the closing patterns of cafes based on the significant global clustering test with a *p*-value of 0.002.

### (3) Comparing the densities of closed and opened restaurants after the pandemic outbreak

**Figure 5:**
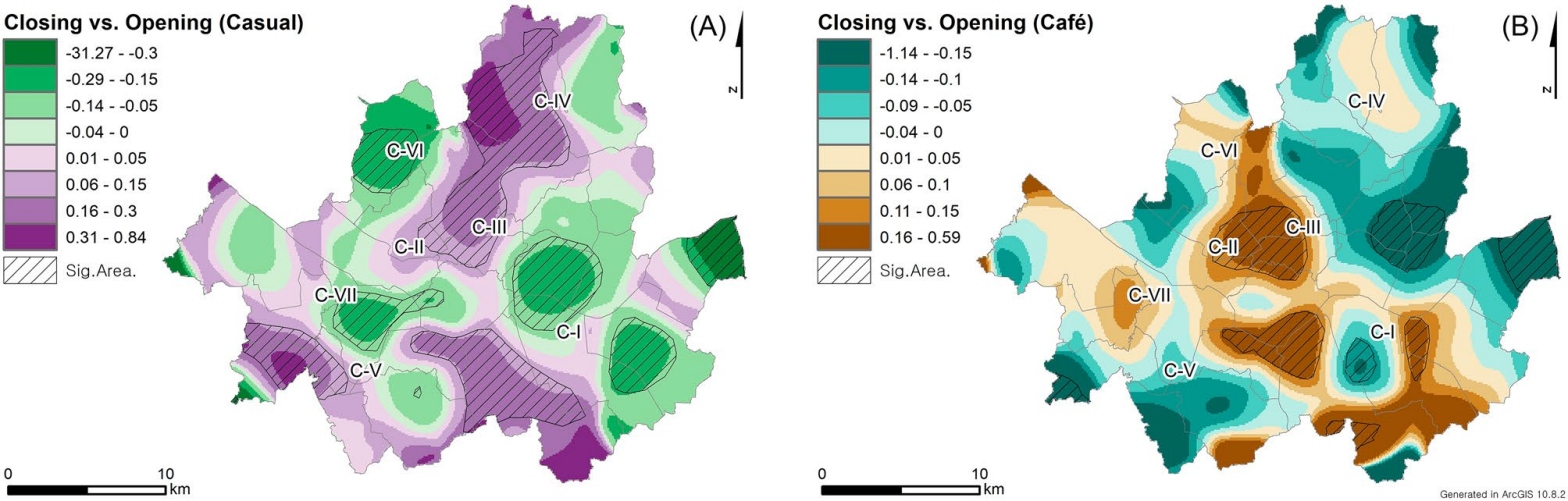
The RRS results by comparing closed and opened (**A**) casual restaurants and (**B**) cafes.

The impacts of the COVID-19 outbreak on the districts of casual restaurants and cafes are examined using RRS by comparing the densities of opened and closed restaurants (Fig. 5). A district with significantly higher densities of closed restaurants than opened restaurants could indicate a decline in restaurant businesses in the district. However, it is important to note that high densities of closed restaurants in a district may not necessarily indicate a decline in local commercial district if the district also has high densities of opening restaurants.

The RRS analysis compares the densities between closed and opened casual restaurants to assess the impacts of the COVID-19 pandemic on local casual restaurant businesses. Although the total number of opened casual restaurants (26,126) in Seoul is greater than that of closed casual restaurants (23,941) during the pandemic period (2020–2021), the RRS result indicate statistically significant local variations in the impacts of the pandemic on casual restaurants (*p*-value = 0.005 in the global clustering test). For instance, C-I and C-IV in Fig. 4A show similar trends with both exhibiting increases in the densities of closed casual restaurants after the outbreak. However, their RRS values in Fig. 5A show opposite trends with C-I showing a negative RRS value (i.e., higher densities in opened casual restaurants) and C-IV showing a positive RRS value (i.e., higher densities in closed casual restaurants). This indicates that the casual restaurant business in C-IV has declined, while that in C-I has been maintained or even improved after the outbreak. Similar patterns are observed in C-III and C-VI, which exhibit trends similar to those of C-IV and C-I, respectively.

The RRS analysis also examines the impacts of the pandemic on cafes by comparing the densities of closed cafes to those of opened cafes (Fig. 5B). Similar to casual restaurant business, the total number of opened cafes (11,985) is slightly higher than that of closed cafes (9980). However, the RRS result also reveals significant local variations in the impacts of the pandemic on cafes (*p*-value = 0.008 in the global clustering test). For example, in Fig. 4B, C-I and C-III both show a decrease in closed café densities after the outbreak, but Fig. 5B reveals distinct patterns in the two clusters. C-I not only exhibits a decrease in closed café densities, but also higher densities of opened cafes than closed cafes, which indicates that this district has not been negatively impacted by the pandemic. In contrast, C-III displays a significant decline in café business with closed café densities remaining higher than those of opened cafes. Similarly, C-VII also shows a higher density of closed cafes (although not statistically significant) than opened cafes along with an increase in densities after the outbreak, which indicates a potential decline in café business in this district.

**Figure 6:**
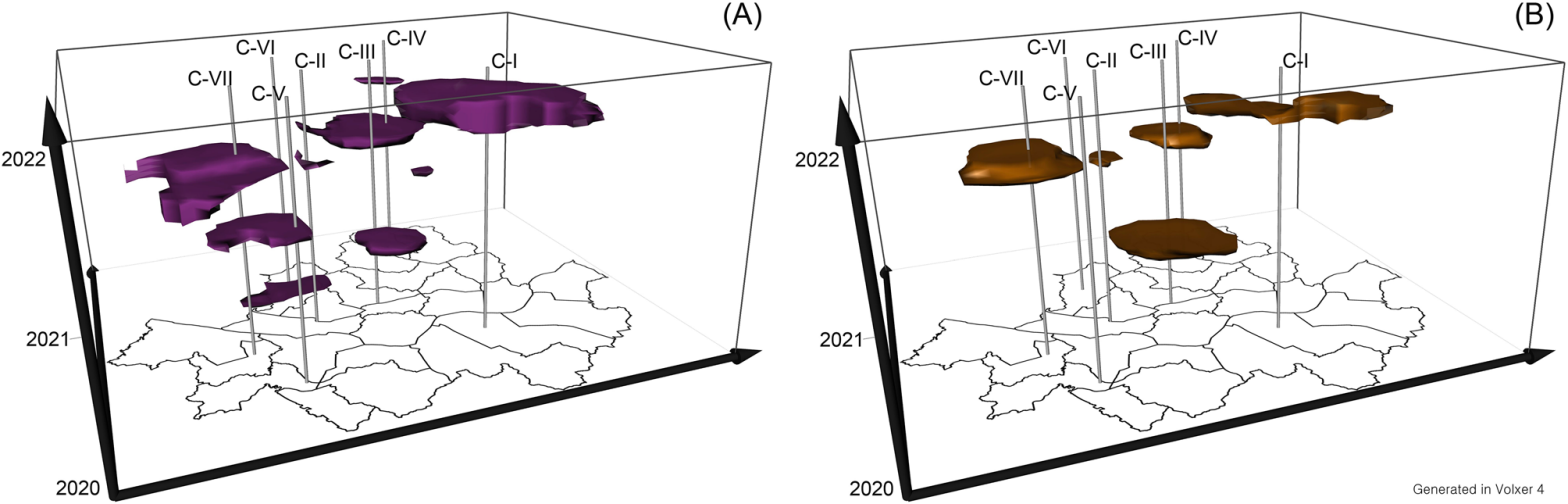
The spatio-temporal clusters obtained from STRRS results by comparing closed and opened (**A**) casual restaurants and (**B**) cafes.

By comparing the spatio-temporal densities of closed and opened restaurants, STRRS identifies regions and time periods of decline in restaurant businesses after the pandemic outbreak. This study estimates the optimal sizes of spatio-temporal bandwidths for STRRS using the plug-in method and adjusted them to 2000 m and 90 days for spatial and temporal bandwidths, respectively, for ease of interpretation. Figure 6 displays the study area (i.e., Seoul) on the x- and y-axes and the temporal dimension from January 1, 2020 to December 31, 2021 on the z-axis. The significant spatio-temporal clusters are represented in isosurfaces^18^, which specifically indicate significantly higher densities of closed restaurants than opened restaurants at the 1% significance level.

The STRRS analysis compares the spatio-temporal densities of closed and opened casual restaurants to identify spatio-temporal clusters of closed restaurants (Fig. 6A). The corresponding RRS result (Fig. 5A) successfully captures the district that show a decline in casual business after the pandemic outbreak. Furthermore, the STRRS result provides specific periods for these declines. For instance, C-IV and C-V show similar RRS results (Figs. 4A and 5A), and the spatio-temporal clusters (Fig. 6A) also occur at a similar period, i.e., January 2021. The RRS result for the southern region of C-I is also similar to that of C-IV and C-V, but its spatio-temporal cluster, i.e., a statistically higher density of closed casual restaurants than opened ones, occurs in December 2021.

Figure 6B shows spatio-temporal clusters of closed cafes using the STRRS analysis by comparing the densities of closed and opened cafes. The isosurfaces based on the STRRS results identify five spatio-temporal clusters, including C-IV and C-VII. Both C-IV and C-VII exhibit declines in café businesses after the pandemic outbreak with similar RRS values (Figs. 4B and 5B), but they occur during slightly different time periods. Specifically, the spatio-temporal clusters in C-IV and C-VII are occurred in March and June 2021, respectively. Similar to the STRRS results for the southern region of C-I in the casual restaurant counterpart, the STRRS results for the café counterpart also reveal spatio-temporal clusters in November 2021.

## Conclusion

This study presents an effective exploration of spatio and spatio-temporal clusters of closed restaurants in Seoul after the COVID-19 pandemic and highlights the utility of RRS extensions for the cluster exploration. First, this study examines the spatial clusters of closed restaurants using KDE, but it fails to effectively identify clusters that deviate from random chances due to inadequate consideration of their underlying population at risk. Second, this study extends RRS and STRRS by incorporating different combinations of cases and control functions, e.g., types of restaurants, times of closures, and closed and opened restaurants. These extensions enable more comprehensive exploration of closed restaurant clusters.

Performing RRS and STRRS with various controls helps to detect significant clusters of closed restaurants by providing additional insights of the clusters. Specifically, the extensions of RRS and STRRS yields the following findings. First, the impacts of the COVID-19 pandemic on local restaurant businesses vary according to the types of restaurants, i.e., casual restaurants and cafes, which suggests the need to examine pandemic impacts by restaurant type. Second, the RRS method allows for estimation of changes in the densities of closed restaurants by comparing their densities before and after the pandemic outbreak. The results reveal regions that have experienced increases in densities (e.g., C-I and C-IV for casual restaurants and cafes, respectively). Third, the RRS method, which compares densities between closed and opened restaurants, is useful for capturing declines in local restaurant businesses. This result helps to recognize spatial clusters where restaurant businesses have declined from those that have not been negatively impacted by the pandemic. Finally, the STRRS method, which compares spatio-temporal densities of closed and opened restaurants, effectively captures both spatio- and temporal clusters of closed restaurants.

This study would contribute to the literature on spatial data analysis and urban policy support in response to future pandemics. This analysis extends RRS and STRRS to detect spatio-temporal clusters of closed restaurants that are deviated from random occurrences with various pairs of cases and control. The extensions also provide a more comprehensive exploration of the impacts of the pandemic on local businesses. Specifically, the study successfully explores the varying impacts of the pandemic on the types of restaurants and identifies changes in the densities of closed restaurants after the pandemic outbreak. Additionally, the study can identify the decline of restaurant businesses by comparing closed and opened restaurant densities and highlight specific spatio-temporal clusters of these declines. As restaurant businesses are a major industry in Seoul, estimating their damages and decline trends in response to infectious diseases can inform policymakers in preparing support plans.

This study can be extended in the several ways. First, alternative control events could be applicable in RRS and STRRS. For instance, this study compares opened dates of restaurants to their closed dates because both specific dates with their spatial coordinates are easily represented in the form of 3-D point datasets. However, the numbers of operating restaurants could provide alternative control events. Second, this study primarily focuses on detecting clusters of closed restaurants, and did not investigated their main factors contributing to these clusters through confirmatory spatial data analyses (CSDA), such as spatial regressions. The clusters this study finds could not be generated by the pandemic, and thus, an extension to CSDA could provide insight into the factors contributing to these clusters^34,35^. Additionally, estimating difference-in-difference by comparing the changes between 2019 and 2018 with those between 2021 and 2020 could be helpful to eliminate temporal trends^36^. Third, this study estimates spatio-temporal clusters under the first-order separable assumption between space and time, which have been widely utilized in space–time analyses^37,38^. However, an approach to accommodate interaction between space time need to be applied in future studies^39^. Fourth, the analysis results potentially include type I errors due to the multiple-testing problem, and the results can generally be corrected based on Bonferroni and false discovery adjustments^25^. However, the application of these existing statistical and spatial statistical (e.g., Getis and Ord’s method)^40^ correction methods into raster-based analyses warrants through investigation in following studies. Finally, although permanent business closure is generally considered the most negative impact of the pandemic on local businesses, it does not occur immediately in response to the pandemic. Therefore, excluding temporary closings^41^ and examining business sales^3^ could be useful in further investigating the impacts of the pandemic.

### Supplementary Information

Supplementary Figure 1.

## Data Availability

All relevant data are available from the corresponding author upon request.
