# Supplementary figures and images for: Exploring the spatio-temporal clusters of closed restaurants after the COVID-19 outbreak in Seoul using relative risk surfaces
Source: Sci Rep. 2023 Aug 24;13:13889. doi: 10.1038/s41598-023-40937-5 (PMC10449878; doi:10.1038/s41598-023-40937-5)

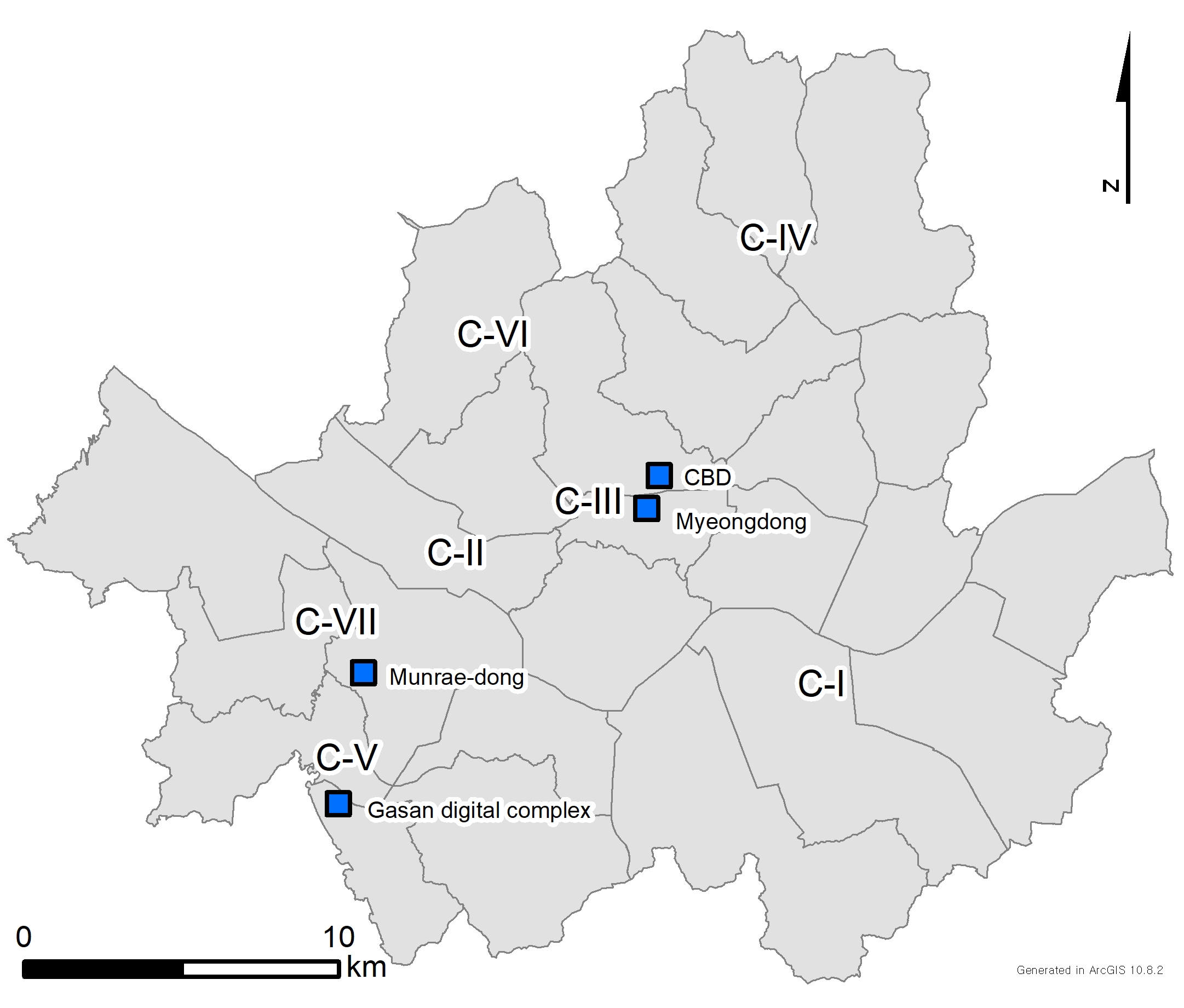

Supplement: Supplementary file 1 — Supplementary Figure 1. [file 41598_2023_40937_MOESM1_ESM.jpg]
